# Autologous chondrocyte implantation-derived synovial fluids display distinct responder and non-responder proteomic profiles

**DOI:** 10.1186/s13075-017-1336-7

**Published:** 2017-06-30

**Authors:** Charlotte H. Hulme, Emma L. Wilson, Mandy J. Peffers, Sally Roberts, Deborah M. Simpson, James B. Richardson, Pete Gallacher, Karina T. Wright

**Affiliations:** 1 Institute of Science and Technology in Medicine, Keele University, Keele, Staffordshire UK; 2 Robert Jones and Agnes Hunt Orthopaedic Hospital, Oswestry, Shropshire UK; 3 Institute of Medicine, Chester University, Chester, UK; 4 Institute of Ageing and Chronic Disease, University of Liverpool, Liverpool, UK; 5 Centre for Proteome Research, Institute of Integrative Biology, University of Liverpool, Liverpool, UK

**Keywords:** Label-free proteomics, Autologous chondrocyte implantation, Synovial fluid

## Abstract

**Background:**

Autologous chondrocyte implantation (ACI) can be used in the treatment of focal cartilage injuries to prevent the onset of osteoarthritis (OA). However, we are yet to understand fully why some individuals do not respond well to this intervention. Identification of a reliable and accurate biomarker panel that can predict which patients are likely to respond well to ACI is needed in order to assign the patient to the most appropriate therapy. This study aimed to compare the baseline and mid-treatment proteomic profiles of synovial fluids (SFs) obtained from responders and non-responders to ACI.

**Methods:**

SFs were derived from 14 ACI responders (mean Lysholm improvement of 33 (17–54)) and 13 non-responders (mean Lysholm decrease of 14 (4–46)) at the two stages of surgery (cartilage harvest and chondrocyte implantation). Label-free proteome profiling of dynamically compressed SFs was used to identify predictive markers of ACI success or failure and to investigate the biological pathways involved in the clinical response to ACI.

**Results:**

Only 1 protein displayed a ≥2.0-fold differential abundance in the preclinical SF of ACI responders versus non-responders. However, there is a marked difference between these two groups with regard to their proteome shift in response to cartilage harvest, with 24 and 92 proteins showing ≥2.0-fold differential abundance between Stages I and II in responders and non-responders, respectively. Proteomic data has been uploaded to ProteomeXchange (identifier: PXD005220). We have validated two biologically relevant protein changes associated with this response, demonstrating that matrix metalloproteinase 1 was prominently elevated and S100 calcium binding protein A13 was reduced in response to cartilage harvest in non-responders.

**Conclusions:**

The differential proteomic response to cartilage harvest noted in responders versus non-responders is completely novel. Our analyses suggest several pathways which appear to be altered in non-responders that are worthy of further investigation to elucidate the mechanisms of ACI failure. These protein changes highlight many putative biomarkers that may have potential for prediction of ACI treatment success.

## Background

Traumatic cartilage injury can lead to the development of osteoarthritis (OA) [1]. Autologous chondrocyte implantation (ACI) has been adopted clinically to repair cartilage damage [2, 3] and is a procedure that involves two surgeries. The first is to harvest cartilage from a minor load-bearing region of the joint (Stage I), followed by a 3–4 week chondrocyte extraction and culture expansion phase, and the second procedure (Stage II) occurs when chondrocytes are implanted into the pathological cartilage defect [3, 4]. Within our centre, the procedure has a 19% failure rate, as defined by a lack of improvement in Lysholm score [5], which is comparable to other centres [6, 7]. Demographic- and injury-associated risk factors for failure have been identified [8–10]; however, currently there is little understanding of the biological nature of ACI failure. We hypothesise that an individual’s probability of failure to respond to ACI can be predicted, and that the metrics required to make such a prediction will come from improved understanding of the pathology of the failure in the first instance. Further, we aim to address the need to identify putative biomarkers that can be used to predict patient long-term outcome prior to cartilage repair therapy. The importance of this has been highlighted by the Osteoarthritis Research Society International (OARSI) who published guidelines highlighting the need “to determine whether biomarkers are useful in identifying those individuals most likely to receive clinically important benefits from an intervention; and to determine whether biomarkers are useful for identifying individuals at earlier stages of OA in order to institute treatment at a time more amenable to disease modification” [11]. The synovial fluid (SF) surrounding the ACI repair site provides a biological fluid that can be assessed to profile the joint environment and to investigate the biological response and innate repair following a ‘controlled injury’, such as that which is sustained at Stage I of the ACI procedure. Despite strong evidence that unbiased proteomic approaches can identify novel biomarkers of OA progression (reviewed in De Ceuninck and Berberbaum [12] and Hsueh et al. [13]), relatively few studies have focused on the SF [14, 15]. The limited numbers of studies that have analysed human SF proteins in cartilage injury and repair have tested for markers of OA of known biological relevance to cartilage injury [16–19]. Using a targeted approach to biomarker identification, our group has been able to demonstrate that the absence of detectable aggrecanase-1 activity in the SF can be used along with lower age and higher baseline knee function as a predictive marker of ACI success [19], indicating that SF biomarkers have the potential for the stratification of patients to appropriate cartilage repair therapies. We are unaware of any published work that has used an untargeted approach to investigate the SF proteome before, during, or after an intervention to treat cartilage injury. There is therefore a requirement to complete an unbiased assessment to identify putative predictive biomarkers that may allow for ACI patient stratification.

The broad dynamic range of proteins in SF [20, 21] means that abundant proteins can make it difficult to interrogate low abundance proteins by suppressing their detectability. The deepest examination of the SF proteome to date (equine SF) [21] used hexapeptide libraries [22, 23] to capture low abundance proteins. In this publication, we have profiled the SF proteome from patients undergoing ACI treatment at both stages of the procedure using dynamic range compression with hexapeptide libraries and label-free quantification proteomics by liquid chromatography/tandem mass spectrometry (LC-MS/MS).

## Methods

### Synovial fluid collection and storage

Following local research ethical committee approval and with informed consent, SF was collected from the knee joints of patients at Stages I (harvest; 15 samples) and II (implantation of cells; 24 samples) of ACI by injecting 20 mL of saline and then extending and flexing the leg at least 20 times prior to intra-articular aspiration of as much SF as possible [24]. SF was then centrifuged at 6000 g for 15 mins at 4 °C before being divided into aliquots and stored in –196 °C liquid nitrogen prior to analyses. Prior to selection of suitable patient samples for this study, the dilution factor of the SFs was assessed by measurement of urea concentrations in the SF and plasma (harvested at the same time). Based on evidence that the urea concentration of plasma and SF is equivalent, the dilution factor could then be calculated as described previously [19, 25]. Any samples with a dilution factor over 10 were excluded from the study.

As has previously been used [26–28], we defined responders as a minimal clinically important difference (MCID) at approximately 12 months post-treatment if they had an increase of 10 points in the Lysholm score (which ranges from 0–100, with 100 representing ‘perfect’ knee function [29]). Fourteen SF donors were considered as responders, with a mean improvement of 33 points (range 17–54) and 13 SF donors were considered as non-responders with a mean decrease in Lysholm score of 14 points (range 4–46). Matched Stage I and II samples were included for seven responders and six non-responders; however, all of the proteomic data presented in this study are based on non-matched statistical comparisons to ensure the maximal number of patient samples could be included in each comparison. The demographic information and change in Lysholm score for these patients is shown in Table 1. None of the demographic parameters, other than difference in Lysholm score between baseline and 12 months post-ACI, were significantly different between responders and non-responders at Stage I or Stage II, or between Stage I and Stage II in responders or non-responders (*p* > 0.05; Mann-Whitney *U* test; Table 1).

**Table 1:** Demographic data for patient participants whose samples from Stage I or Stage II were analysed who responded clinically (responders) or who did not respond (non-responders) to autologous chondrocyte implantation (ACI)

	Stage I		Stage II		*p* value (A) R v NR SI; (B) R v NR SI	*p* value (A) SI v SII R; (B) SI v SII NR
	Responders (*n* = 8)	Non-responders (*n* = 7)	Responders (*n* = 12)	Non-responders (*n* = 12)		
Difference in Lysholm Score	27 (17–38)	–8 (–4 to –17)	34 (17–54)	–11 (–4 to –46)	(A) 0.0003; (B) <0.0001	(A) 0.21; (B) 0.55
BMI (kg/m^2^)	29 (23–31)	27 (24–31)	27 (23–48)	29 (22–36)	(A) 0.94; (B) 0.54	(A) 0.73; (B) 0.68
Age (years)	32 (17–49)	40 (25–50)	40 (17–90)	43 (25–52)	(A) 0.28; (B) 0.92	(A) 0.17; (B) 0.58
Male (*n*)	8	7	11	10	(A) >0.99; (B) >0.99	(A) >0.99; (B) 0.51
Smoker (*n*)	1	2	1	3	(A) 0.54; (B) 0.59	(A) >0.99; (B) >0.99
Dilution factor of SF	5 (3–9)	4 (2–7)	4 (1–9)	3 (2–5)	(A) 0.48; (B) 0.25	(A) 0.53; (B) 0.50
Total defect area (cm^2^)	14 (0.4–24)	6 (0.6–12)	6 (1–20)	5 (0.6–12)	(A) 0.74; (B) 0.35	(A) 0.45; (B) 0.28
Patella defect (*n*)	1	1	4	2	(A) >0.99; (B) 0.64	(A) 0.60; (B) >0.99
LFC defect (*n*)	2	0	0	0	(A) 0.47; (B) >0.99	(A) 0.15; (B) >0.99
LTP defect (*n*)	1	0	0	0	(A) >0.99; (B) >0.99	(A) 0.15; (B) >0.99
MFC defect (*n*)	2	2	1	6	(A) >0.99; (B) 0.07	(A) 0.54; (B) 0.63
Trochlea defect (*n*)	0	3	2	1	(A) 0.20; (B) >0.99	(A) 0.49; (B) 0.12
Multiple defects (*n*)	1	0	1	1	(A) >0.99; (B) >0.99	(A) >0.99; (B) >0.99
Unknown defect location (*n*)	1	1	4	2	(A) >0.99; (B) 0.64	(A) 0.60; (B) >0.99

None of the demographic parameters, other than difference in Lysholm scores, showed differences between responders (*R*) and non-responders (*NR*) in individuals whose synovial fluids (*SFs*) from Stage I (*SI*) or Stage II (*SII*) were compared, nor were there differences between individuals who were either responders or non-responders when comparing Stage I and Stage II samples (*p* ≥ 0.05; Mann-Whitney *U* test). Data are shown as median (range) unless otherwise indicated. *BMI* body mass index, *LFC* lateral femoral condyle, *LTP* lateral tibial plateau, *MFC* medial femoral condyle

### Sample preparation and analysis using label-free proteomics

All SF samples were maintained as separate samples throughout the protein equalisation, mass spectrometry, and label-free quantification steps, and no pooling of any samples was performed; hence, the abundance of proteins was quantified for each of the 39 samples and mean protein abundance across the experimental groups was calculated prior to analysis of protein changes.

#### SF preparation and protein equalisation using ProteoMiner™

The dynamic range of proteins in SF was compressed using ProteoMiner™ beads (BioRad, Hemel Hempstead, UK) as described previously [21]. Briefly, SF was treated with hyaluronidase (1 mg/ml) [21, 30] and digestion was confirmed using a Coomassie stained 1D-SDS PAGE gel. Total protein was quantitated using a Pierce™ 660-nm protein assay (Thermo Scientific, Hemel Hempstead, UK) [31] and 5 mg of total protein was exposed to ProteoMiner™ beads. After washing, bead-bound proteins were treated with 0.05% (w/v) RapiGest (Waters, Manchester, UK) in 25 mM ammonium bicarbonate for 10 min at 80 °C prior to reduction, alkylation, and in-situ protein digestion without removal of the beads, ensuring complete proteome access. The digestion was completed in LoBind protein tubes (Eppendorf, Stevenage, UK) followed by acidification of trifluoroacetic acid to a final concentration of 0.5% (v/v). This treatment inactivates and precipitates the Rapigest detergent which can then be removed by centrifugation. The peptide-containing supernatant fractions were frozen at –20 °C prior to LC-MS/MS.

#### Mass spectrometry and label-free quantification

Tryptic peptides were subjected to LC-MS/MS, analysed using a 2-h gradient on a NanoAcquity™ ultraperformance LC (Waters, Manchester, UK) coupled online to a Q-Exactive Quadrupole-Orbitrap instrument (Thermo-Fisher Scientific Hemel Hempstead, UK) as described previously [32]. For label-free quantification, the raw files of the acquired spectra were analysed by the ProgenesisQI™ software (Waters, Manchester, UK) [33]. Briefly, the top five spectra for each feature were exported from ProgenesisQI™ and utilised for peptide identification with a locally implemented Mascot server (Version 2.3.01), searching against the Unihuman reviewed database. Search parameters used were: peptide mass tolerances, 10 ppm; fragment mass tolerance, 0.01 Da; 2+ and 3+ ions; missed cleavages, 1; enzyme, trypsin; instrument type, ESI-FTICR. Modifications included were: fixed carbamidomethyl cysteine and variable oxidation of methionine, lysine, and proline. To maximise the number of quantifiable proteins but simultaneously use an acceptable false discovery rate (FDR), the peptide matches above an identify-threshold were adjusted to give an FDR of 1% before the protein identifications were re-imported into ProgenesisQI™ for the label-free relative quantification. Quantification was undertaken using unique peptides only. Statistical analysis was performed using ProgenesisQI™ software; briefly transformed normalised abundances were used for one-way analysis of variance (ANOVA) and all peptides (with *p* < 0.05) of an identified protein were included. For analysis of the proteins, the mean abundance of each protein across the experimental groups (e.g. Stage I samples from responders etc.) was calculated and those proteins with a ≥2.0-fold-change (FC) between the comparator groups were reported. For use in network and pathway analysis, however, a less stringent cut-off of ≥1.2-FC was used to allow for the study of systemic changes, as has been appropriate in similar studies using pathway and network approaches [34].

### Pathway and network analysis of proteomic datasets

Proteins were analysed using the pathway enrichment and topological analysis tools in Ingenuity™ (Qiagen, US) [35] to identify and visualise the canonical pathways which are differentially affected between Stages I and II of ACI. To allow for greater confidence in the pathway analysis, an independent platform, the Database for Annotation, Visualization and Integrated Discovery (DAVID) [36], was used to analyse functional pathway based on the protein changes.

### Validation of mass spectrometry using ELISA

Two proteins were selected to validate the MS findings because they: (1) had associated biological relevance to cartilage injury and repair; (2) showed consistent differential abundance between Stages I and II of ACI in non-responders versus responders; and (3) could be measured using available enzyme-linked immunosorbent assays (ELISAs). These proteins, matrix metalloproteinase 1 (MMP1) [37] and S100 calcium binding protein A13 (S100-A13) [38], were quantified using duo-set ELISAs (R&D systems, Wiesbardon, Germany) according to the manufacturer’s instructions. Samples were assessed in duplicate and mean optical density values were used to calculate the protein concentration. SF was diluted 1:2 and 1:100 for the assessment of MMP1 and S100-A13, respectively. The protein concentration of each protein was normalised to total protein concentration. Statistical analysis was performed in GraphPad Prism version 6.0.

## Results

Proteomic data has been deposited in the PRIDE ProteomeXchange and can be accessed using the identifier PXD005220 [39].

### Differential abundance of proteins in responders versus non-responders to ACI

**Fig. 1:**
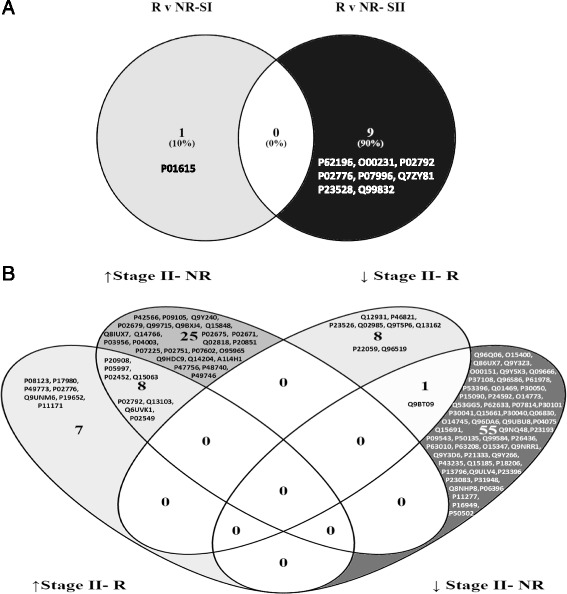
Venn-Diagrams representing the proteins identified using label-free quantification proteomics which were differentially abundant (≥2.0-FC) in the SF (**a**) at Stage I (*SI*) or Stage II (*SII*) in responders (*R*) compared to non-responders (*NR*) to ACI, (**b**) showing increased (↑) or decreased (↓) abundance at Stage II compared to stage I of ACI in clinical responders (R) or non-responders (NR)

Proteomic analysis identified that Ig kappa chain V-II region MIL demonstrated a +2.6-FC in non-responders versus responders to ACI in SF at Stage I (Fig. 1a). Nine proteins demonstrated differential abundance at Stage II between responders and non-responders (26S protease regulatory subunit 7, +2.3-FC; 26S proteasome non-ATPase regulatory subunit 13, +2.4-FC; ferritin light chain, +2.9-FC; platelet factor 4, +3.3-FC; thrombospondin-1, +3.4-FC; nucleosome assembly protein 1-like 1, +4.9-FC; cofilin-1, +7.1-FC; EH domain-containing protein 1, +7.3-FC; and T-complex protein 1 subunit eta, +8.4-FC) (Fig. 1a).

### Differential abundance of proteins after controlled cartilage injury (Stage I versus Stage II)

When comparing Stage I with Stage II, 116 proteins were >2.0-fold differentially abundant. Non-responders to ACI displayed a distinct and marked response to Stage I surgery, such that between Stages I and II, 33 proteins were upregulated and 59 downregulated, 12 of which demonstrated common expression change in clinical responders to ACI (Table 2; Fig. 1b). Fifteen proteins were upregulated and nine proteins were downregulated between Stage I and Stage II in responders to ACI (Table 3; Fig. 1b).

**Table 2:** Fold-change of proteins that are differentially expressed in the synovial fluid collected at Stage I compared to Stage II of the ACI procedure in clinical non-responders. Proteins shown in italic were validated using enzyme linked immunosorbant assay

Protein		Fold change
Description	Accession	
Perilipin-4	Q96Q06	–4.3
Syntaxin-7	O15400	–3.9
Fermitin family homolog 3	Q86UX7	–3.7
Deoxynucleoside triphosphate triphosphohydrolase SAMHD1	Q9Y3Z3	–3.7
PDZ and LIM domain protein 1	O00151	–3.6
Sorting nexin-5	Q9Y5X3	–3.3
Neuroblast differentiation-associated protein AHNAK	Q09666	–3.2
Signal recognition particle 14 kDa protein	P37108	–3.0
Hyaluronan and proteoglycan link protein 3	Q96S86	–3.0
Heterogeneous nuclear ribonucleoprotein K	P61978	–2.9
ATP-citrate synthase	P53396	–2.9
Fatty acid-binding protein, epidermal	Q01469	–2.8
60S ribosomal protein L12	P30050	–2.7
Fatty acid-binding protein, adipocyte	P15090	–2.6
Insulin-like growth factor-binding protein 6	P24592	–2.6
Protein canopy homolog 3	Q9BT09	–2.6
Tripeptidyl-peptidase 1	O14773	–2.5
PDZ and LIM domain protein 3	Q53GG5	–2.5
Cellular nucleic acid-binding protein	P62633	–2.5
Bifunctional glutamate/proline-tRNA ligase	P07814	–2.5
Protein disulfide-isomerase A3	P30101	–2.4
Peroxiredoxin-6	P30041	–2.4
Tryptase alpha/beta-1	Q15661	–2.4
Endoplasmic reticulum resident protein 29	P30040	–2.4
Peroxiredoxin-1	Q06830	–2.4
Na(+)/H(+) exchange regulatory cofactor NHE-RF1	O14745	–2.4
Mitochondrial import inner membrane translocase subunit TIM14	Q96DA6	–2.4
Mortality factor 4-like protein 1	Q9UBU8	–2.4
Fructose-bisphosphate aldolase A	P04075	–2.3
Microtubule-associated protein RP/EB family member 1	Q15691	–2.3
Leucine zipper transcription factor-like protein 1	Q9NQ48	–2.3
Transcription elongation factor A protein 1	P23193	–2.3
2',3'-cyclic-nucleotide 3'-phosphodiesterase	P09543	–2.3
Histamine N-methyltransferase	P50135	–2.3
*Protein S100*-*A13*	Q99584	–2.3
Acrosomal protein SP-10	P26436	–2.2
AP-2 complex subunit beta	P63010	–2.2
S-phase kinase-associated protein 1	P63208	–2.2
High mobility group protein B3	O15347	–2.2
Cytokine-like protein 1	Q9NRR1	–2.2
Mitochondrial fission 1 protein	Q9Y3D6	–2.2
Filamin-A	P21333	–2.2
Nuclear migration protein nudC	Q9Y266	–2.1
Cathepsin K	P43235	–2.1
Prostaglandin E synthase 3	Q15185	–2.1
Vinculin	P18206	–2.1
Plastin-2	P13796	–2.1
Coronin-1C	Q9ULV4	–2.1
Ig heavy chain V-I region V35	P23083	–2.1
Stress-induced-phosphoprotein 1	P31948	–2.1
Putative phospholipase B-like 2	Q8NHP8	–2.1
Gelsolin	P06396	–2.0
Spectrin beta chain, erythrocytic	P11277	–2.0
Stathmin	P16949	–2.0
Hsc70-interacting protein	P50502	–2.0
40S Ribosomal protein	P23396	–2.0
Epidermal growth factor receptor substrate 15	P42566	22.5
Hemoglobin subunit theta-1	P09105	6.1
C-type lectin domain family 11 member A	Q9Y240	5.6
Periostin	Q15063	5.1
Collagen alpha-1(I) chain	P02452	4.7
Spectrin alpha chain, erythrocytic 1	P02549	4.0
Collagen alpha-1(V) chain	P20908	3.7
Fibrinogen gamma chain	P02679	3.5
Collagen alpha-1(XII) chain	Q99715	3.3
Complement C1q tumor necrosis factor-related protein 3	Q9BXJ4	3.1
Adiponectin	Q15848	3.0
Adipocyte enhancer-binding protein 1	Q8IUX7	3.0
Latent-transforming growth factor beta-binding protein 1	Q14766	3.0
Fibrinogen beta chain	P02675	3.0
Chondroitin sulfate proteoglycan 4	Q6UVK1	2.9
Fibrinogen alpha chain	P02671	2.8
*Interstitial collagenase* (*MMP1*)	P03956	2.7
Secreted phosphoprotein 24	Q13103	2.7
Collagen alpha-2(V) chain	P05997	2.6
Ferritin light chain	P02792	2.6
C4b-binding protein alpha chain	P04003	2.6
Nucleobindin-1	Q02818	2.4
C4b-binding protein beta chain	P20851	2.2
Vitamin K-dependent protein S	P07225	2.2
Fibronectin	P02751	2.2
Prosaposin	P07602	2.1
Integrin beta-like protein 1	O95965	2.1
Adipocyte plasma membrane-associated protein	Q9HDC9	2.1
Cytoplasmic dynein 1 heavy chain 1	Q14204	2.0
Soluble scavenger receptor cysteine-rich domain-containing protein SSC5D	A1L4H1	2.0
F-actin-capping protein subunit beta	P47756	2.0
Mannan-binding lectin serine protease 1	P48740	2.0
Thrombospondin-3	P49746	2.0

Positive numbers denote an increase in the protein at Stage II; negative numbers denote a decrease in the protein at Stage II

**Table 3:** Proteins that are differentially expressed with a ≥2.0-fold-change in the synovial fluid collected at Stage I compared to Stage II of the ACI procedure in clinical responders

Protein		Fold change
Description	Accession	
Heat shock protein 75 kDa, mitochondrial	Q12931	–470.0
Microtubule-associated protein 1B	P46821	–17.2
Adenosylhomocysteinase	P23526	–3.7
Complement factor H-related protein 3	Q02985	–2.8
Mannose-1-phosphate guanyltransferase beta	Q9Y5P6	–2.7
Peroxiredoxin-4	Q13162	–2.5
Oxysterol-binding protein 1	P22059	–2.4
Protein canopy homolog 3	Q9BT09	–2.1
Spermatid perinuclear RNA-binding protein	Q96SI9	–2.0
Ferritin light chain	P02792	2.2
Secreted phosphoprotein 24	Q13103	2.2
Chondroitin sulfate proteoglycan 4	Q6UVK1	2.3
Collagen alpha-2(I) chain	P08123	2.3
Collagen alpha-1(V) chain	P20908	2.5
26S protease regulatory subunit 6A	P17980	2.7
Collagen alpha-2(V) chain	P05997	2.9
Collagen alpha-1(I) chain	P02452	3.3
Spectrin alpha chain, erythrocytic 1	P02549	4.1
Histidine triad nucleotide-binding protein 1	P49773	4.2
Platelet factor 4	P02776	4.2
Alpha-1-acid glycoprotein 2	P19652	4.3
Protein 4.1	P11171	4.3
Periostin	Q15063	4.5
26S proteasome non-ATPase regulatory subunit 13	Q9UNM6	4.6

Positive numbers denote an increase in the protein at Stage II, negative numbers denote a decrease in the protein at Stage II

### Identification of canonical pathways and protein networks associated with protein changes between Stage I and II

**Fig. 2:**
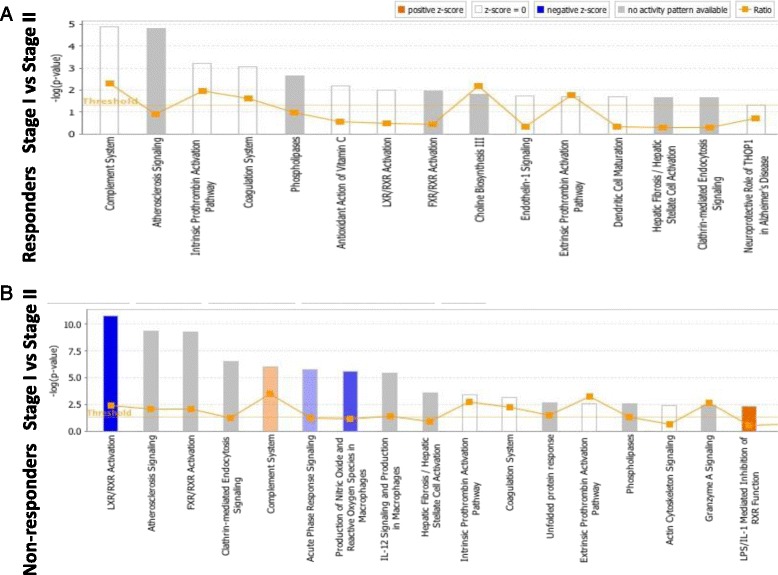
Canonical pathways altered in the synovial fluid of responders (**a**) and non-responders (**b**) at Stage I compared to Stage II of ACI, identified using Ingenuity analysis, based on proteins which were identified using label-free quantification proteomics (≥1.2-FC). The *bars* represent the significance of the canonical pathway as calculated by a right-sided Fisher’s exact test; therefore, the tallest bars represent the canonical pathways that are the least likely to have been identified due to molecules being in the canonical pathway by random chance. Canonical pathways which are likely activated (based on the pattern of differentially abundant proteins) are shown in *orange* and pathways that are likely inhibited are shown in *blue*

**Fig. 3:**
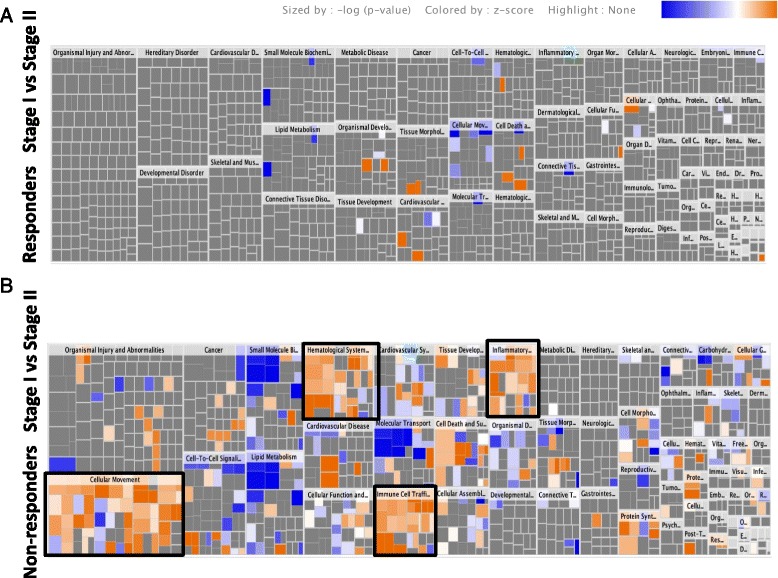
Heat map showing canonical pathway groupings for molecular and cellular functions altered in the synovial fluid of responders (**a**) and non-responders (**b**) at Stage I compared to Stage II of ACI, identified using Ingenuity analysis, based on proteins which were identified using label-free quantification proteomics (≥1.2-FC). *Squares* are coloured based on their *z* score, with *orange* being up at Stage II and *blue* being down at Stage II; the colour intensity indicates the prediction strength. The *z* score represents whether the up- or downregulation of the proteins within that function will lead to activation (positive *z* score) or inhibition (negative *z* score) of the function. *Black boxes* are shown around functions of biological interest: cellular movement, haematological system development and function, immune cell trafficking, and inflammatory response

Several canonical pathways were associated with the protein changes identified in both clinical responders and non-responders (Fig. 2). Figure 3, however, highlights the disparity in the proteome response to Stage I between clinical responders and non-responders, as a much greater number of functional/disease pathways were activated or inhibited in association with these differentially abundant proteins. Using Ingenuity software, canonical pathways that were most significantly connected with these protein changes in non-responders were Liver X Receptor/Retinoic X receptor (LXR/RXR) activation (*p* = 1.63 × 10^–7^), complement system (*p* = 9.33 × 10^–7^), and acute phase response signalling (*p* = 1.69 × 10^–6^) (Fig. 2). Independent pathway analysis of the proteins using DAVID also highlighted the complement system (*p* = 2.1 × 10^–6^) as highly associated with the protein changes seen between Stages I and II of ACI in non-responders.

**Fig. 4:**
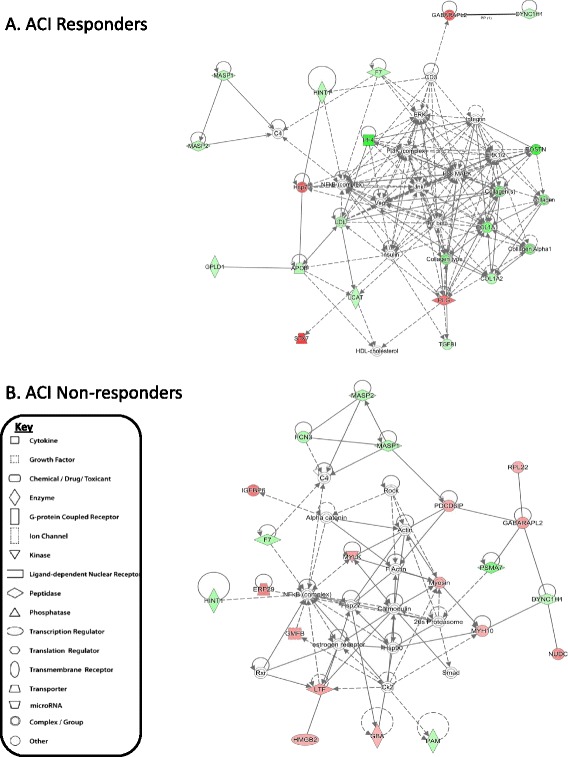
Top scoring networks derived from the proteins with different abundance (≥1.2-FC) at Stage II compared to Stage I of the autologous chondrocyte implantation (*ACI*) procedure in those who responded well clinically (responders; **a**) and those who did not respond clinically (non-responders; **b**). *Red* nodes represent greater protein abundance at Stage II of ACI; *green* nodes represent lower protein abundance at Stage II of ACI; and *white* nodes represent inferred proteins which are not differentially expressed between Stage I and Stage II (based on label-free quantification proteomic analysis). The key to the features within the network is shown. *APOB* Apolipoprotein B-100, *CD3* T-cell surface glycoprotein CD3, *Ck2* Casein kinase 2, *COL1A1* Collagen alpha-1(I) chain, *COL1A2* Collagen alpha-2(II) chain, *C4* Complement C4, *DYNC1H1* Cytoplasmic dynein 1 heavy chain 1, *ERK* Mitogen-activated protein kinase 3, *ERP29* Endoplasmic reticulum resident protein 29, *F7* Coagulation factor VII, *FCN3* Ficolin-3, *GABRAPL2* Gamma-aminobutyric acid receptor-associated protein-like 2, *GBA* Glucosylceramidase, *GMFB* Glia maturation factor beta, *GPLD1* Phosphatidylinositol-glycan-specific phospholipase D, *HINT1* Histidine triad nucleotide-binding protein 1, *HDL*-*cholesterol* High density lipoprotein-cholesterol, *HMGB2* High mobility group protein B2, *HSP76* Heat shock protein 76, *IGFBP6* Insulin-like growth factor binding protein 6, *Jnk* Mitogen-activated protein kinase, *LCAT* Phosphatidylcholine-sterol acyltransferase, *LDL* Low-density lipoprotein receptor, *LTF* Lactotransferrin, *MASP1* Mannan-binding lectin serine protease 1, *MASP2* Mannan-binding lectin serine protease 2, *MYH10* Myosin-10, *MYLK* Myosin light chain kinase, smooth muscle, *NFkB complex* nuclear factor kappa-light-chain-enhancer of activated B cells complex, *PAM* Peptidyl-glycine alpha-amidating monooxygenase, *PDCD6IP* Programmed cell death 6-interacting protein, *PF4* Platelet factor 4, *PI3K complex* Phosphoinositide-3 kinase complex, *PLG* Plasminogen, *POSTN* periositin, *PSMA7* Proteasome subunit alpha type-7, *P38 MAPK* P38 mitogen-activated protein-kinases, *Rock* Rho-associated protein kinase 1, *RPL22* 60S ribosomal protein L22, *Rxr* retinoic X receptor, *STX7* Syntaxin-7, *Tgf* beta Transforming growth factor beta, *Vegf* Vascular endothelial growth factor

The Stage I versus Stage II responder network consisted predominantly of proteins associated with connective tissue disorders (*p* = 8.2 × 10^–7^). In non-responders to ACI, however, the top scoring network included several proteins associated with the inflammatory response (*p* = 2.75 × 10^–5^) (Fig. 4). Transforming growth factor beta 1 (TGFβ1) and CCAAT/enhancer-binding protein beta were predicated to be the most significant upstream regulators associated with the networks of protein changes identified between Stages I and II in non-responders and responders, respectively (Table 4). TGFβ was also predicted as an upstream regulator of the protein changes identified between Stages I and II in responders; therefore, it may be important in the regulation of protein response between Stages I and II, irrespective of outcome.

**Table 4:** Analysis of upstream regulators of interactome networks generated from protein changes between Stages I (SI) and II (SII) in either responders or non-responders to ACI were identified using Ingenuity Pathway Analysis software

Upstream regulators		Activation *z* score (SII v SI)	*p* value of overlap	Target molecules in dataset
Non-responders				
	TGFB1	–1.595	9.46E–10	APOB,APOC2,APOE,CD44,COL1A1,COL1A2,COL5A1,COMP,CSPG4,CTSD,ECM1,FETUB,FN1,FTL,GSN,HINT1,HSPG2,HTRA1,IGFBP6,LCAT,MYLK,PCOLCE2,PDXK,POSTN,RAP1A,S100A4,TGFBI
	DYSF	NP	2.01E–09	CFD,FN1,FTL,LCP1,LYZ,PROS1,S100-A13,S100A4
	MYC	3.046	2.20E–09	ALDOA,ANXA5,CCT3,CD44,COL1A1,COL1A2,COL5A1,CSPG4,CTSD,ECM1,FN1,HSPA9,LYZ,NCL,NUCB1,NUDC,PAM,PTN,RPL22,RPL30,TF
	COL9A1	1.308	2.79E–09	COMP,FN1,HSPG2,TGFBI,THBS4
	Beta-estradiol	2.271	5.01E–09	ALDOA,APOE,CD44,COL1A1,COL1A2,COMP,CTSD,F7,FN1,GMFB,HSPA2,HSPA8,HSPA9,HTRA1,IGFBP6,LTF,LYZ,MYLK,PAM,PDIA3,QSOX1,RAP1A,RPS13,S100-A13,SLC9A3R1,TF,THBS4
	Lipopolysaccharide	–0.104	5.96E–09	ANXA5,APOB,APOC2,APOE,CD44,CFD,COL1A1,COL1A2,COL5A1,CSPG4,FN1,GSN,HDGFRP3,HMGB2,HSPA8,HTRA1,ITIH2,LBP,LTF,LYZ,PARK7,PCOLCE,PCOLCE2,PDIA3,PLG,TF
	Dihydrotestosterone	–1.091	1.03E–08	ALDOA,APOE,CCT3,FN1,FTL,GSN,HINT1,LYZ,MYLK,NUCB1,PAM,POSTN,PROS1,RPL30,TF
	HRAS	0.623	2.52E–08	CD44,COL1A1,COL1A2,ERP29,FN1,GSN,HSPA8,HTRA1,LYZ,MYH10,PDIA5,PLTP,POSTN,RPL30,S100A4
	KRAS	2.226	3.99E–08	ALDOA,CD44,COL1A1,FN1,GBA,GSN,MYLK,PCOLCE,PDIA3,PSMA7,RNASE4,S100A4
	SMARCB1	–1.195	4.82E–08	APOC4,CD44,COL1A1,COL1A2,GSN,LBP,POSTN,PTN,RAB14
Responders				
	CEBPB	–1.067	2.03E–07	APOB,CFD,COL1A1,COL1A2,F7,HSPA8,PLG
	FLI1	NP	3.82E–07	COL1A1,COL1A2,HSPA8,PF4
	S-adenosylhomocysteine	NP	1.21E–06	COL1A1,COL1A2
	SCX	NP	1.65E–06	COL1A1,COMP,POSTN
	Tgf beta (group)	–1.454	5.16E–06	COL1A1,COL1A2,LCAT,POSTN,TGFBI
	ENTPD5	NP	1.21E–05	COL1A1,COL1A2
	MKX	NP	1.21E–05	COL1A1,COL1A2
	GATA4	NP	2.86E–05	COL1A1,COL1A2,POSTN,TGFBI
	Nilotinib	NP	3.39E–05	COL1A1,COL1A2
	TBX5	NP	5.50E–05	COL1A1,COL1A2,POSTN

The 10 upstream regulators with the lowest *p* values are demonstrated for both responders and non-responders. The *p* value of overlap is calculated based on the overlap between protein changes within the dataset with known targets of the transcriptional regulator, calculated using a Fisher’s exact test. The activation *z* score can be used to infer likely activation states of the upstream regulators based on the direction of protein abundance change in the dataset, i.e. a negative activation *z* score indicates that the upstream regulator is downregulated at Stage II compared to stage I, thus eliciting the specific directions of protein changes of the target molecules at Stage II compared to Stage I of ACI. *NP* indicates no prediction of activation status could be generated by the software

### Measurement of MMP1 and S100-A13 protein in SF by ELISA

**Fig. 5:**
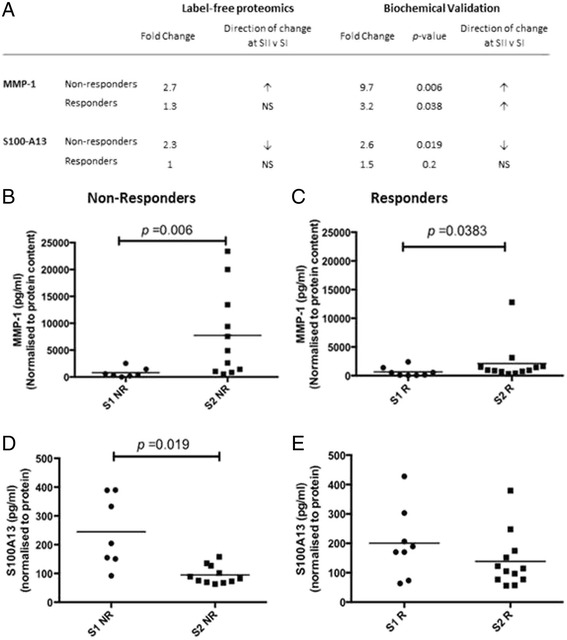
Two biologically relevant proteins, matrix metalloproteinase-1 (*MMP1*) and S100 calcium binding protein A13 (*S100*-*A13*), that were identified by proteomic analysis as differentially abundant in the SF of non-responders between Stages I (*SI*) and II (*SII*) of the *ACI* procedure were validated by ELISA. **a** The differential abundance as measured by label-free mass-spectrometry and by biochemical ELISA. MMP1 was measured by ELISA in the SF of (**b**) non-responders (*NR*) and (**c**) responders (*R*) to ACI at cartilage harvest (Stage I; *S1*) and chondrocyte implantation (Stage II; *S2*). S100-A13 was measured by ELISA in the SF of non-responders (**d**) and responders (**e**) to ACI

MMP1 and S100-A13 protein abundance in SF from the same patient cohort was measured using ELISA. Biochemical assessment replicated the MS finding that SF MMP1 concentration is significantly increased at Stage II in non-responders (Stage I, 800 ± 889 pg/ml; Stage II, 7741 ± 8065 pg/ml (mean ± SD); *p* = 0.006; Mann-Whitney *U* test) (Fig. 5). However, measurement of MMP1 via ELISA also demonstrated a significant increase in concentration at Stage II compared to Stage I in responders to ACI (albeit to a lesser order of magnitude: Stage I, 655 ± 837 pg/ml; Stage II, 2672 ± 3576 pg/ml (mean ± SD); *p* = 0.039; Mann-Whitney *U* test) (Fig. 5). In this patient cohort, no correlation between Lysholm score and MMP1 could be demonstrated (*r* = 0.02; *p* = 0.94; Spearman’s correlation).

Biochemical quantification identified a significant decrease in S100-A13 expression at Stage II (94 ± 31 pg/ml (mean ± SD)) compared to Stage I (245 ± 123 pg/ml (mean ± SD); *p* =0.02; Mann-Whitney *U* test) of the ACI process in clinical non-responders and no significant difference in responders (Stage I, 200 ± 118 (mean ± SD); Stage II, 138 ± 93 (mean ± SD); *p* = 0.2; Mann-Whitney *U* test) thus validating MS findings (Fig. 5). To assess whether the differential abundance of S100-A13 identified between Stages I and II was genuinely only a response of clinical non-responders, further statistical analysis including only the paired Stage I (*n* = 7) and II (*n* = 6) samples was completed. The paired analysis also confirmed the MS findings, with non-responders having lower SF S100-A13 concentration at Stage II compared to Stage I (Stage I, 270 ± 113 (mean ± SD); Stage II, 116 ± 112 (mean ± SD); *p* = 0.03; Wilcoxon-matched pairs) and no significant difference in S100-A13 concentration between Stages I and II demonstrated in clinical responders (Stage I, 204 ± 127 (mean ± SD); Stage II, 140 ± 53 (mean ± SD); *p* = 0.22; Wilcoxon-matched pairs). Again, Lysholm score and S100-A13 concentration did not correlate in this cohort (*r* = 0.0002; *p* = 0.99; Spearman’s correlation).

## Discussion

The unbiased quantitative study of the human SF proteome is relatively limited [14, 15] and few studies are reported which identify novel biomarkers for the diagnosis of cartilage injury or for their prognostic or predictive value in the treatment of cartilage injuries [16, 18]. To our knowledge, only four studies have assessed SF or blood for biomarkers of cartilage injury treatment [16, 17, 19, 40] from which limited putative predictive markers have been identified.

The patient cohort used in this study presents an opportunity to explore which proteins are altered between individuals who have responded well to ACI and those non-responders whose joint function has deteriorated post-treatment. Although a non-responder is generally defined as an individual who does not demonstrate an improvement in Lysholm score of 10 or more points, we have been able to identify proteins that relate to the worst clinical outcomes in our cohort; in future studies, these protein changes will need to be explored further in non-responders with a lesser negative response to ACI. It would have been interesting to assess how cartilage regeneration related to clinical outcome in these patients; unfortunately, however, data regarding the quality and amount of repair tissue in the defect site post-treatment were not routinely collected for these patients.

Ideally, a biomarker that predicts response to cell therapy would be measured before the patient undergoes cartilage harvest. This study, however, demonstrated that only one protein displayed differential abundance between responders and non-responders at Stage I of the ACI procedure. Interestingly, the vast majority of differences in the proteomic profile of SF were evident between Stages I and II of the ACI procedure, particularly in non-responders to ACI. Analysis of the pathways that are altered between Stages I and II of ACI illustrates further the disparity in biological response to a controlled cartilage injury in clinical responders versus non-responders. This suggests that it may be the response of the patients to the cartilage harvest procedure at Stage I that proves to be the best predictor of clinical outcome.

One of the biologically relevant proteins identified that increased in the SF of non-responders at Stage II compared to Stage I of ACI was MMP1. Along with other MMP family members, it is overexpressed in many forms of arthritis [37] and has been strongly associated with increased joint inflammation [41, 42]. Since MMP1 has cartilage degradation properties [37], implanting chondrocytes into a joint with high levels of active MMP1 might be detrimental to any new cartilage formation. However, the ELISA used in this study assessed total MMP1 protein; therefore, additional studies to determine how much active MMP1 is present would be needed to further elucidate the mechanisms of action. Nonetheless, a higher absolute level of MMP1 found in the SF of non-responders at Stage II compared to Stage I of the ACI procedure is suggested to be predictive of a poor outcome, which could inform the second stage of the ACI procedure. For example, it may be that an individual with high levels of MMP1 at Stage II requires a tailored procedure and could benefit from delayed chondrocyte implantation to allow for dampening of inflammation or a coincidental treatment to reduce MMP1 activity specifically or to reduce joint inflammation.

Interestingly, S100-A13 abundance was found to be significantly lower in the SF from non-responders to ACI at Stage II compared to Stage I. This finding is completely novel since, despite six of these family members having been studied in cartilage (S100B [43], S100-A2 [44], S100-A4 [45], S100-A8 [46], S100-A9 [46], and S100-A11 [47]), we are unaware of any studies that have assessed the role of S100-A13 in cartilage or SF. S100-A13 therefore presents an attractive novel candidate for further study, not only to confirm its potential as a predictive biomarker but also to improve our biological understanding of the processes underlying cartilage injury and repair.

Despite MMP1 and S100-A13 both having potential as candidate biomarkers to determine whether or not a patient is suitable to continue to the second stage of ACI, it is unlikely that any individual biomarkers will be sufficient to determine which patients are suitable candidates for a cartilage repair therapy. Patient demographics have already been identified which are known to pre-dispose to ACI failure, including gender, body mass index, age, and the size of the cartilage lesion [10, 25, 48]. Our long-term aim is to work towards the development of a clinical prediction model which will likely include known risk factors, along with a panel of biomarkers that, together, can predict the response of an individual to ACI. This exploratory study has indicated a plethora of potential SF biomarkers that may contribute to the development of such a clinical prediction model. Other studies have also indicated that the quality of the culture-expanded autologous chondrocytes affects patient clinical outcome (higher percentage positivity of CD44-expressing cells and increased collagen type II and aggrecan expression correlate with good clinical outcome, whereas lower cell viability correlates with poor clinical response to ACI) [28, 49, 50]. Therefore, ideally, the quality of ACI cells prior to implantation at Stage II, along with known demographic risk factors, would be considered as part of a clinical prediction matrix. We have already shown that the absence of detectable aggrecanse-1 is able to predict ACI success, and that lower age and the use of a collagen patch are also indicators of ACI success [19]; consequently, markers identified within this study may be added to this developing predictive model.

Many of the protein shifts observed in the SF between Stages I and II of the ACI procedure are likely to be associated with acute mechanisms of cartilage healing in response to the cartilage harvest, which could be considered as a controlled injury. Furthermore, the differences observed in this shift when comparing responders and non-responders to ACI could be a result of differences in the mechanisms of action and/or the rate/magnitude of an individual’s innate capacity for cartilage healing. Our evaluation of the SF proteome shift both before and after such a defined injury represents a period of acute response to injury and could therefore be considered as a model of short-term natural healing in humans. In order to validate that the response observed in this model is due to the cartilage harvest and not the arthroscope procedure, an appropriate control group should be evaluated. In future work we will aim to test potential biomarkers in a stem cell treatment group which will similarly undergo a Stage I arthroscope (but without a cartilage harvest) followed by cell implantation at Stage II in a comparable time frame.

Pathway and network analyses were performed to try and elucidate the functional implications of the observed proteome shift. Confidence can be taken from these findings, as pathways of known biological relevance were identified. For example, the complement system, which was activated in non-responders to ACI, can lead to cartilage degradation [47]. Other biologically relevant pathways that were identified include the acute phase response, which in contrast was dysregulated in non-responders. Dysregulation of the acute phase response has previously been associated with the proteome of OA knees [51], and serum amyloid-A, a key acute-phase protein, is increased in abundance in the SF and blood of individuals with OA [52]. Both of these observations indicate that the acute-phase response may be a contributing factor as to why some individuals do not respond well to ACI and there is merit in further studying how this pathway is altered in these individuals.

Interestingly, TGFβ1 was suggested as the most potent potential upstream regulator of the network of non-responder SF protein response to ACI. TGFβ1 is suggested to regulate twenty-seven of the network proteins, and therefore manipulation of this biological network via TGFβ1 may serve as a potential method to influence response to ACI in clinical non-responders. It is strongly established that TGFβ signalling is highly influential on the development and progression of OA [53]. Specifically, TGFβ is important in the regulation of chondrocyte hypertrophy and maturation [54]. Mutations in the TGFβ1 gene, as well as genetic variation in other members of the TGFβ signalling pathway, have been related to OA development [53]. Moreover, in transgenic animals which overexpress the type-II TGFβ receptor, chondrocytes in the superficial zone of the cartilage are hypertrophic with increased type X collagen expression and decreased proteoglycans [55]. Together, these observations highlight the importance of TGFβ for chondrocyte homeostasis; therefore, in non-responders to ACI, perhaps aberrant upstream expression of TGFβ contributes to their poor cartilage formation.

Canonical pathway analysis has also highlighted more novel pathway modifications within non-responders to ACI such as inhibition of LXR/RXR. Dimerisation of RXRs and LXR initiates transcriptional regulation that is involved in the regulation of inflammation [56]. Interestingly, proteomic profiling of the SF from osteoarthritic shoulders has highlighted dysregulation of the LXR/RXR pathway in response to OA [57], and agonism of the LXR/RXR pathway has previously been suggested as a therapeutic target for OA since human cartilage explants treated with a synthetic LXR agonist showed reduced cytokine-mediated degradation of proteoglycans [58]. Although further study is required to determine how inhibition of the LXR/RXR pathway may lead to a poor clinical response to ACI, it may be that these individuals are demonstrating a more ‘osteoarthritic’ phenotype, perhaps meaning that ACI may be insufficient to repair their cartilage injury.

The most striking biologically relevant canonical pathway groupings that were upregulated in non-responders to ACI were inflammatory and immune responses. This strengthens the suggestion that poor response to ACI in these individuals may be attributed to the chondrocytes being implanted into an unsuitable, highly inflammatory environment. Alternatively, it may indicate that these individuals have a more pronounced immune reaction to cartilage injury in general, and that this is an indicative response of ‘poor healers’.

## Conclusion

We have identified proteins that are altered within the SF following cartilage injury and ACI, and have highlighted proteome changes in response to the cartilage harvest procedure in ACI which relates to clinical outcome. These protein changes represent a plethora of potential predictive biomarkers that, with further validation, could help in the identification of patients who are not suited to ACI or perhaps any cartilage repair procedure. Pathway and network analyses of the altered SF proteome have highlighted both known and novel biological pathways that may be implicated in the response to Stage I of ACI. These data present an opportunity for future study which could vastly improve our knowledge of how a joint responds to cartilage injury and how that may differ between individuals who do or do not have the capacity for cartilage repair.

## Abbreviations

ACI: Autologous chondrocyte implantation
ANOVA: Analysis of variance
APOB: Apolipoprotein B-100
C4: Complement C4
CD3: T-cell surface glycoprotein CD3
Ck2: Casein kinase 2
COL1A1: Collagen alpha-1(I) chain
COL1A2: Collagen alpha-2(II) chain
DAVID: Database for Annotation, Visualization and Integrated Discovery
DYNC1H1: Cytoplasmic dynein 1 heavy chain 1
ELISA: Enzyme-linked immunosorbent assay
ERK: Mitogen-activated protein kinase 3
ERP29: Endoplasmic reticulum resident protein 29
F7: Coagulation factor VII
FC: Fold-change
FCN3: Ficolin-3
FDR: False discovery rate
GABRAPL2: Gamma-aminobutyric acid receptor-associated protein-like 2
GBA: Glucosylceramidase
GMFB: Glia maturation factor beta
GPLD1: Phosphatidylinositol-glycan-specific phospholipase D
HDL: High-density lipoprotein
HINT1: Histidine triad nucleotide-binding protein 1
HMGB2: High mobility group protein B2
HSP76: Heat shock protein 76
IGFBP6: Insulin-like growth factor binding protein 6
Jnk: Mitogen-activated protein kinase
LCAT: Phosphatidylcholine-sterol acyltransferase
LC-MS/MS: Liquid chromatography tandem mass-spectrometry
LDL: Low-density lipoprotein receptor
LTF: Lactotransferrin
LXR/RXR: Liver X Receptor/Retinoic X receptor
MASP1: Mannan-binding lectin serine protease 1
MASP2: Mannan-binding lectin serine protease 2
MCID: Minimal clinically important difference
MMP1: Matrix metalloproteinase 1
MYH10: Myosin-10
MYLK: Myosin light chain kinase, smooth muscle
NFkB complex: Nuclear factor kappa-light-chain-enhancer of activated B cells complex
OA: Osteoarthritis
OARSI: Osteoarthritis Research Society International
P38 MAPK: P38 mitogen-activated protein-kinases
PAM: Peptidyl-glycine alpha-amidating monooxygenase
PDCD6IP: Programmed cell death 6-interacting protein
PF4: Platelet factor 4
PI3K complex: Phosphoinositide-3 kinase complex
PLG: Plasminogen
POSTN: Periositin
PSMA7: Proteasome subunit alpha type-7
Rock: Rho-associated protein kinase 1
RPL22: 60S ribosomal protein L22
Rxr: Retinoic X receptor
S100-A13: S100 calcium binding protein A13
SF: Synovial fluid
STX7: Syntaxin-7
Tgf beta: Transforming growth factor beta
TGFβ1: Transforming growth factor beta 1
Vegf: Vascular endothelial growth factor

## References

[1] Lotz MK, Kraus VB (2010). New developments in osteoarthritis. Posttraumatic osteoarthritis: pathogenesis and pharmacological treatment options. Arthritis Res Ther.

[2] Gillogly SD, Voight M, Blackburn T (1998). Treatment of articular cartilage defects of the knee with autologous chondrocyte implantation. J Orthop Sport Phys Ther.

[3] Richardson JB, Caterson B, Evans EH, Ashton BA, Roberts S (1999). Repair of human articular cartilage after implantation of autologous chondrocytes. J Bone Joint Surg Br.

[4] Wright KT, Mennan C, Fox H, Richardson JB, Banerjee R, Roberts S (2013). Characterization of the cells in repair tissue following autologous chondrocyte implantation in mankind: a novel report of two cases. Regen Med.

[5] Lysholm J, Gillquist J (1982). Evaluation of knee ligament surgery results with special emphasis on use of a scoring scale. Am J Sports Med.

[6] Knutsen G, Drogset JO, Engebretsen L, Grontvedt T, Ludvigsen TC, Loken S (2016). A randomized multicenter trial comparing autologous chondrocyte implantation with microfracture: long-term follow-up at 14 to 15 years. J Bone Jt Surg Am.

[7] Niemeyer P, Porichis S, Steinwachs M, Erggelet C, Kreuz PC, Schmal H (2014). Long-term outcomes after first-generation autologous chondrocyte implantation for cartilage defects of the knee. Am J Sports Med.

[8] Dugard MN, Herman Kuiper J, Parker J, Roberts S, Robinson E, Harrison P (2017). Development of a tool to predict outcome of autologous chondrocyte implantation. Cartilage.

[9] Niemeyer P, Salzmann GM, Hirschmüller A, Südkamp NP (2012). Factors that influence clinical outcome following autologous chondrocyte implantation for cartilage defects of the knee. Z Orthop Unfall.

[10] Jungmann PM, Salzmann GM, Schmal H, Pestka JM, Sudkamp NP, Niemeyer P (2012). Autologous chondrocyte implantation for treatment of cartilage defects of the knee: what predicts the need for reintervention?. Am J Sports Med.

[11] Kraus VB, Blanco FJ, Englund M, Henrotin Y, Lohmander LS, Losina E (2015). OARSI Clinical Trials Recommendations: soluble biomarker assessments in clinical trials in osteoarthritis. Osteoarthr Cartil.

[12] De Ceuninck F, Berenbaum F (2009). Proteomics: addressing the challenges of osteoarthritis. Drug Discov Today.

[13] Hsueh MF, Onnerfjord P, Kraus VB (2014). Biomarkers and proteomic analysis of osteoarthritis. Matrix Biol.

[14] Liao W, Li Z, Wang H, Wang J, Fu Y, Bai X (2013). Proteomic analysis of synovial fluid: Insight into the pathogenesis of knee osteoarthritis. Int Orthop.

[15] Liao W, Li Z, Zhang H, Li J, Wang K, Yang Y (2015). Proteomic analysis of synovial fluid as an analytical tool to detect candidate biomarkers for knee osteoarthritis. Int J Clin Exp Pathol.

[16] Vasara AI, Konttinen YT, Peterson L, Lindahl A, Kiviranta I (2009). Persisting high levels of synovial fluid markers after cartilage repair: a pilot study. Clin Orthop Relat Res.

[17] Wasilko SM, Tourville TW, DeSarno MJ, Slauterbeck JR, Johnson RJ, Struglics A (2016). Relationship between synovial fluid biomarkers of articular cartilage metabolism and the patient’s perspective of outcome depends on the severity of articular cartilage damage following ACL trauma. J Orthop Res.

[18] Watt FE, Paterson E, Freidin A, Kenny M, Judge A, Saklatvala J (2016). Acute molecular changes in synovial fluid following human knee injury. Arthritis Rheumatol.

[19] Wright KT, Kuiper JH, Richardson JB, Gallacher P, Roberts S. The absence of detectable ADAMTS-4 (aggrecanase-1) activity in synovial fluid is a predictive indicator of autologous chondrocyte implantation success. Am J Sports Med. 2017. doi:10.1177/0363546517694027. 10.1177/0363546517694027 28277753

[20] Gibson DS, Rooney ME (2007). The human synovial fluid proteome: a key factor in the pathology of joint disease. Proteomics-Clinical Applications.

[21] Peffers MJ, McDermott B, Clegg PD, Riggs CM (2015). Comprehensive protein profiling of synovial fluid in osteoarthritis following protein equalization. Osteoarthr Cartil.

[22] Hartwig S, Czibere A, Kotzka J, Passlack W, Haas R, Eckel J (2009). Combinatorial hexapeptide ligand libraries (ProteoMiner): an innovative fractionation tool for differential quantitative clinical proteomics. Arch Physiol Biochem.

[23] Freeby S, Walker J, Paulus A, Smith K, Liu N, Academia K. Enrichment of medium- and low-abundance proteins in sample types using ProteoMiner technology. 2010:1–6.

[24] Roberts S, Evans H, Wright K, van Niekerk L, Caterson B, Richardson JB (2015). ADAMTS-4 activity in synovial fluid as a biomarker of inflammation and effusion. Osteoarthritis Cartilage.

[25] Kraus V, Stabler T, Kong S, Varjum G, McDaniel G (2007). Measurement of synovial fluid volume using urea. Osteoarthr Cartil.

[26] Ehrich E, Davies G, Watson D, Bolohnese J, Seidenberg B, Bellamy N (2000). Minimal perceptible clinical improvement with the Western Ontario and McMaster Universities osteoarthritis index questionnaire and global assessments in patients with osteoarthritis. J Rheumatol.

[27] Roos E, Lohmander L (2003). The Knee Injury and Osteoarthritis Outcome Score (KOOS): from joint injury to osteoarthritis. Health Qual Life Outcomes.

[28] Saris D, Vanlauwe J, Victor J, Almqvist K, Verdonk R, Bellemans J (2009). Treatment of symptomatic cartilage defects in the knee: characterized chondrocyte implantation results in better clinical outcome at 36 months in a randomized trial compared to microfracture. Am J Sports Med.

[29] Smith H, Richardson J, Tennant A (2009). Modification and validation of the Lysholm Knee Scale to assess articular cartilage damage. Osteoarthr Cartil.

[30] Mateos J, Lourido L, Fernández-Puente P, Calamia V, Fernández-López C, Oreiro N, Ruiz-Romero C, Blanco FJ (2012). Differential protein profiling of synovial fluid from rheumatoid arthritis and osteoarthritis patients using LC–MALDI TOF/TOF. J Proteomics.

[31] Stoscheck CM (1987). Protein assay sensitive at nanogram levels. Anal Biochem.

[32] Thorpe CT, Peffers MJ, Simpson D, Halliwell E, Screen HRC, Clegg PD (2016). Anatomical heterogeneity of tendon: fascicular and interfascicular tendon compartments have distinct proteomic composition. Sci Rep.

[33] Peffers MJ, Beynon RJ, Clegg PD (2013). Absolute quantification of selected proteins in the human osteoarthritic secretome. Int J Mol Sci.

[34] Su Y, Zhang Y (2015). Identification of biological processes and genes for gestational diabetes mellitus. Arch Gynecol Obstet.

[35] Ingenuity Qiagen. Ingenuity Knowledge Base. 2014.

[36] Sherman BT, Huang DW, Tan Q, Guo Y, Bour S, Liu D (2007). DAVID Knowledgebase: a gene-centered database integrating heterogeneous gene annotation resources to facilitate high-throughput gene functional analysis. BMC Bioinformatics.

[37] Murphy G (1995). Matrix metalloproteinases and their inhibitors. Acta Orthop Scand Suppl.

[38] Yammani RR (1822). S100 proteins in cartilage: role in arthritis. Biochim Biophys Acta Mol Basis Dis.

[39] Vizcaino JA, Csordas A, Del-Toro N, Dianes JA, Griss J, Lavidas I (2016). 2016 update of the PRIDE database and related tools. Nucleic Acids Res.

[40] Nganvongpanit K, Pothacharoen P, Chaochird P, Klunklin K, Warrit K, Settakorn J (2009). Prospective evaluation of serum biomarker levels and cartilage repair by autologous chondrocyte transplantation and subchondral drilling in a canine model. Arthritis Res Ther.

[41] Yoshihara Y, Nakamura H, Obata K, Yamada H, Hayakawa T, Fujikawa K (2000). Matrix metalloproteinases and tissue inhibitors of metalloproteinases in synovial fluids from patients with rheumatoid arthritis or osteoarthritis. Ann Rheum Dis.

[42] Tchetverikov I, Lohmander LS, Verzijl N, Huizinga TWJ, TeKoppele JM, Hanemaaijer R (2005). MMP protein and activity levels in synovial fluid from patients with joint injury, inflammatory arthritis, and osteoarthritis. Ann Rheum Dis.

[43] Wolff DA, Stevenson S, Goldberg VM (1992). S-100 protein immunostaining identifies cells expressing a chondrocytic phenotype during articular cartilage repair. J Orthop Res.

[44] Balmain N, Moutahir F, Heizmann CW, Lieberherr M (2003). Immunolocalization of S100A2 calcium-binding protein in cartilage and bone cells. Cell Mol Biol (Noisy-le-Grand).

[45] Hopwood B, Tsykin A, Findlay DM, Fazzalari NL (2007). Microarray gene expression profiling of osteoarthritic bone suggests altered bone remodelling, WNT and transforming growth factor-beta/bone morphogenic protein signalling. Arthritis Res Ther.

[46] Zreiqat H, Howlett CR, Gronthos S, Hume D, Geczy CL (2007). S100A8/S100A9 and their association with cartilage and bone. J Mol Histol.

[47] Cecil DL, Johnson K, Rediske J, Lotz M, Schmidt AM, Terkeltaub R (2005). Inflammation-induced chondrocyte hypertrophy is driven by receptor for advanced glycation end products. J Immunol.

[48] Jaiswal PK, Bentley G, Carrington RW, Skinner JA, Briggs TW (2012). The adverse effect of elevated body mass index on outcome after autologous chondrocytes implantation. J Bone Jt Surg.

[49] Pietschmann MF, Horng A, Niethammer T, Pagenstert I, Sievers B, Jansson V (2009). Cell quality affects clinical outcome after MACI procedure for cartilage injury of the knee. Knee Surg Sport Traumatol Arthrosc.

[50] Niemeyer P, Pestka JM, Salzmann GM, Sudkamp NB, Schmal H (2012). Influence of cell quality on clinical outcome after autologous chondrocyte implantation. Am J Sports Med.

[51] Ritter SY, Subbaiah R, Bebek G, Crish J, Scanzello CR, Krastins B (2013). Proteomic analysis of synovial fluid from the osteoarthritic knee: Comparison with transcriptome analyses of joint tissues. Arthritis Rheum.

[52] de Seny D, Cobraiville G, Charlier E, Neuville S, Esser N, Malaise D (2013). Acute-phase serum amyloid A in osteoarthritis: regulatory mechanism and proinflammatory properties. PLoS One.

[53] Shen J, Li S, Chen D (2014). TGF-beta signaling and the development of osteoarthritis. Bone Res.

[54] Yang X, Chen L, Xu X, Li C, Huang C, Deng CX (2001). TGF-beta/Smad3 signals repress chondrocyte hypertrophic differentiation and are required for maintaining articular cartilage. J Cell Biol.

[55] Serra R, Johnson M, Filvaroff EH, LaBorde J, Sheehan DM, Derynck R (1997). Expression of a truncated, kinase-defective TGF-beta type II receptor in mouse skeletal tissue promotes terminal chondrocyte differentiation and osteoarthritis. J Cell Biol.

[56] Germain P, Chambon P, Eichele G, Evans RM, Lazar MA, Leid M (2006). Retinoid X receptors. Pharmacol Rev.

[57] Wanner J, Subbaiah R, Skomorovska-Prokvolit Y, Shishani Y, Boilard E, Mohan S (2013). Proteomic profiling and functional characterization of early and late shoulder osteoarthritis. Arthritis Res Ther.

[58] Collins-Racie LA, Yang Z, Arai M, Li N, Majumdar MK, Nagpal S (2009). Global analysis of nuclear receptor expression and dysregulation in human osteoarthritic articular cartilage. Reduced LXR signaling contributes to catabolic metabolism typical of osteoarthritis. Osteoarthr Cartil.

